# *Antrodia salmonea* Extracts Regulate p53-AR Signaling and Apoptosis in Human Prostate Cancer LNCaP Cells

**DOI:** 10.1155/2022/7033127

**Published:** 2022-11-29

**Authors:** Chieh-Yin Chen, Yu-Hsuan Li, Wan-Ling Liao, Muhammet Oner, Yu-Chiao Cheng, Fang-Ling Liu, Pang-Ting Cheng, Ayse Celik, Jyh-Horng Wu, Chih-Ho Lai, Jer-Tsong Hsieh, Ho Lin, Ting-Chieh Chang, Chih-Ying Chang, Mei-Chih Chen

**Affiliations:** ^1^The Experimental Forest, College of Bio-Resources and Agriculture, National Taiwan University, Nantou 55750, Taiwan; ^2^Translational Cell Therapy Center, Department of Medical Research, China Medical University Hospital, Taichung 40447, Taiwan; ^3^Department of Life Sciences, National Chung Hsing University, Taichung 40227, Taiwan; ^4^Department of Forestry, National Chung Hsing University, Taichung 40227, Taiwan; ^5^Department of Microbiology and Immunology, Graduate Institute of Biomedical Sciences, Chang Gung University, Taoyuan 33302, Taiwan; ^6^Department of Urology, University of Texas Southwestern Medical Center, Dallas, Texas 75390, USA

## Abstract

*Antrodia salmonea* (AS) is a genus of *Antrodia*, an epiphyte of Cunninghamia konishii in Taiwan. AS has been reported to have potential therapeutic effects on different diseases, including diarrhea, abdominal pain, and hypertension. AS has been reported to have anticancer effects on numerous cancer types, such as ovarian carcinoma and triple-negative breast cancer. Our previous studies demonstrated that antrocins and triterpenoids are possibly bioactive compositions. However, the effects of AS on prostate cancer remain unknown. Therefore, we investigated the role of AS in prostate cancer growth, apoptosis, and cell cycle regulation. The results showed that AS extracts significantly inhibited the proliferation of prostate cancer LNCaP cells in a dose-dependent manner and increased the levels of apoptotic markers (cleaved PARP and cleaved caspase 3/8/9). In addition, the cell cycle-related proteins CDK1, CDK2, CDK4, and their respective specific regulators Cyclin B1, Cyclin A, and Cyclin D were also affected. Besides, AS treatment increased p53 protein levels and slowed its degradation in LNCaP cells. Interestingly, we found that AS treatment reduced both total protein and Ser-81 phosphorylation levels of the androgen receptor (AR). Notably, the increase of nuclear p53 was accompanied by the down-regulation of AR, suggesting a reverse regulation between p53 and AR in LNCaP cells was triggered by AS treatment. These findings suggest that AS extracts trigger the apoptosis of prostate cancer cells through the reverse regulation of p53 and AR and elucidate that AS extracts might be a potential treatment for androgen-dependent prostate cancer in the near future.

## 1. Introduction

The androgen receptor (AR) is a ligand-dependent transcription factor that belongs to the nuclear steroid hormone receptor family. AR is mainly activated by androgens, including 5*α*-dihydrotestosterone (DHT) and testosterone. AR activation by androgen binding participates in male puberty development and adult reproductive function while maintaining sexual desire and spermatogenesis in adult males [1, 2]. The classical AR transactivation occurs when androgens bind to its ligand-binding domain (LBD). Conformational changes in LBD induced by androgens trigger AR dimerization and phosphorylation at serine or tyrosine residues. Subsequently, the dimerized AR translocates into the nucleus and binds to the specific binding site of the androgen responsive element (ARE) to promote the expression of target genes [3, 4]. The AR activation state needs to maintain a unique balance in healthy individuals. It is known that the imbalance of the AR activation state is the main cause of the development of prostate cancer [4].

Prostate cancer is a highly malignant tumor and the second leading cause of cancer death in men [5]. In the early stage, androgens and AR are required to maintain the proliferation and migration of prostate cancer cells. Therefore, androgen deprivation therapy (ADT) is one of the effective strategies utilized for early prostate cancer or accompanied by surgical resection of tumor tissue [6]. In our previous study, we reported that Cyclin-dependent kinase 5 (CDK5), a unique member of Cyclin-dependent kinases, regulates AR activation during the development of prostate cancer [4, 7–12]. In addition, it is known that the tumor suppressor gene p53, also a cell cycle regulator, can negatively regulate the cell growth of prostate cancer [13, 14]. Moreover, p53 can negatively regulate AR expression in prostate cancer cells [15]. Taken together, targeting cell cycle-related regulators as well as AR is a promising therapeutic approach in the treatment of androgen-dependent prostate cancer cells.

*Antrodia salmonea* (AS) is a fungus belonging to a fungal family endemic in Taiwan with the features of antioxidant, anti-inflammatory, and anticancer. AS extracts have been used to relieve adverse reactions caused by diseases, including diarrhea, abdominal pain, hypertension, and itchy skin [8, 16]. It has been reported that AS extracts suppress the progression of breast cancer by inducing cell cycle arrest and inducing apoptosis in ovarian cancer [8, 17]. Our previous studies have demonstrated the comparison of bioactivity between the extracts of AS and *Antrodia cinnamomea* (AC) and indicated that the secondary metabolites including antcins, triterpenoids, benzenoids, and ubiquinol derivatives are all potential compositions [18–20]. Although the bioactivity of AS extract has been identified and the potential compositions addressed, the effect of AS extract on prostate cancer remains unknown.

In this study, we hypothesize that AS can potentially inhibit prostate cancer cell growth through the regulation of p53-dependent AR inhibition. Therefore, an androgen-dependent human prostate cancer cell line, LNCaP, was used as an *in vitro* cell model to investigate the potential therapeutic effects of AS on human prostate cancer. We demonstrated that AS extracts inhibited cell viability through suppression of the expression and activation of AR protein as well as the activation of nuclear p53 in LNCaP cells. In addition, AS-induced apoptosis was observed in LNCaP cells while reducing protein levels of particular cell cycle regulators. Our findings suggest that AS extract regulates p53-AR and thus induces apoptosis in prostate cancer cells, and is an effective treatment for androgen-dependent human prostate cancer.

## 2. Materials and Methods

### 2.1. Cell Culture and Chemicals

LNCaP is an androgen-sensitive prostate cancer cell line. All cell lines were purchased from Bioresource Collection and Research Center (BCRC) in Taiwan. LNCaP were maintained in RPMI medium 1640 (Gibco, 31800-022, USA) supplemented with 10% fetal bovine serum (FBS, Gibco, 10437, USA), 1 mM sodium pyruvate (SP, Gibco, 11360–070, USA), and 1% penicillin/streptomycin (P/S, Gibco, 15140–122, USA). All cells were incubated at 37°C with 5% CO_2_ and passaged every 3 to 4 days. R1881 (methyltrienolone, a synthetic androgen) was purchased from BIOTANG Inc.; cycloheximide (protein synthesis inhibitor) was purchased from Sigma-Aldrich.

### 2.2. Ethanol Extract of AS Fruiting Body

The protocol for the preparation of the ethanol extract of AS fruiting bodies was described previously [21, 22]. Briefly, the AS fruiting bodies were dried for 48–72 hours in 37°C and AS were ground to powder, accurately weighed (around 48.57 g), placed in an Erlenmeyer flask with 500 mL 95% EtOH, and sonicated in an ultrasonicator for 30 min twice. The extracts were then decanted, filtered under vacuum, concentrated in a rotary evaporator at 50°C, and lyophilized. AS extracts can be dissolved in DMSO.

### 2.3. Cell Viability Analysis

The treated cells were trypsinized and mixed with the completed medium, followed by a 300 × g centrifugation for 5 minutes. The pellet was suspended in PBS and cell proliferation ability was measured by Trypan blue assay.

### 2.4. Cell Protein Extraction and Western Blot Analysis

Experiments were performed as previously described [23]. Briefly, after treatment, the collected cells were lysed in lysis buffer for 45 minutes on ice, followed by 15,400 g and 20 minutes of centrifugation to obtain protein extract. The protein lysate was quantified by Bradford reagent (Sigma-Aldrich, St. Louis, MO, USA). The protein sample was separated by 10% SDS-PAGE. After transferring the protein to PVDF membranes (PerkinElmer Life Sciences, Shelton, CT, USA), which were blocked with 5% skim milk and then incubated with primary antibodies at 4°C overnight. After washing the PVDF membrane with PBST, the membrane was incubated with horseradish peroxidase (HRP)-conjugated secondary antibodies (Jackson Immuno Research Laboratory, West Grove, PA, USA) at room temperature for 1 hour. The Enhanced Chemiluminescence (PerkinElmer Life Sciences, Shelton, CT, USA) reaction was performed, and the membranes were exposed to ChemiDoc (BIO-RED, Berkeley, CA, USA). Antibodies used for detecting target protein included p53 (sc-126, Santa Cruz), p21 (#2947, cell signaling), CDK1 (sc-54, Santa Cruz), CDK2 (sc-163, Santa Cruz), CDK4 (GTX102993, GeneTex), Cyclin A (sc-239, Santa Cruz), Cyclin B1 (#4139, cell signaling), Cyclin E1 (GTX103045, GeneTex), cleaved PARP (AB3565, Millipore), cleaved caspase 3 (#AB3623, Millipore), cleaved caspase 8 (#9496, Cell signaling), cleaved caspase 9 (#52873, cell signaling), p-S81-AR (07-1375, Millipore), AR (441, Santa Cruz), PARP (sc-8007, Santa Cruz), Tubulin (05–829, Millipore), GAPDH (GTX100118, GeneTex), and Actin (MAB1501, Millipore).

### 2.5. shRNA Transfection

To knockdown specific proteins, small hairpin RNAs, shGFP (TRCN0000072192) and shTP53 (TRCN0000003756), were obtained from the Nation RNAi Core Facility of Academia Sinica, Taiwan. The shRNA was diluted in Opti-MEM (Gibco) and premixed with Lipofectamine 3000 and P3000 transfection reagents (invitrogen) according to the manufacturer's instructions. Then the mixture, which is a liposome/nucleic acids complex, was transfected into the LNCaP cells for 48 hours.

### 2.6. Statistical Analysis

Data were presented as the mean ± S.E.M. (Standard error of the mean) and paired Student's *t*-test was used to calculate the *p*-value. Statistical significance was marked as ^*∗*^*p* < 0.05, ^∗∗^*p* < 0.01, and ^∗∗∗^*p* < 0.001. No significance was marked as n.s.

## 3. Results and Discussion

**Figure 1:**
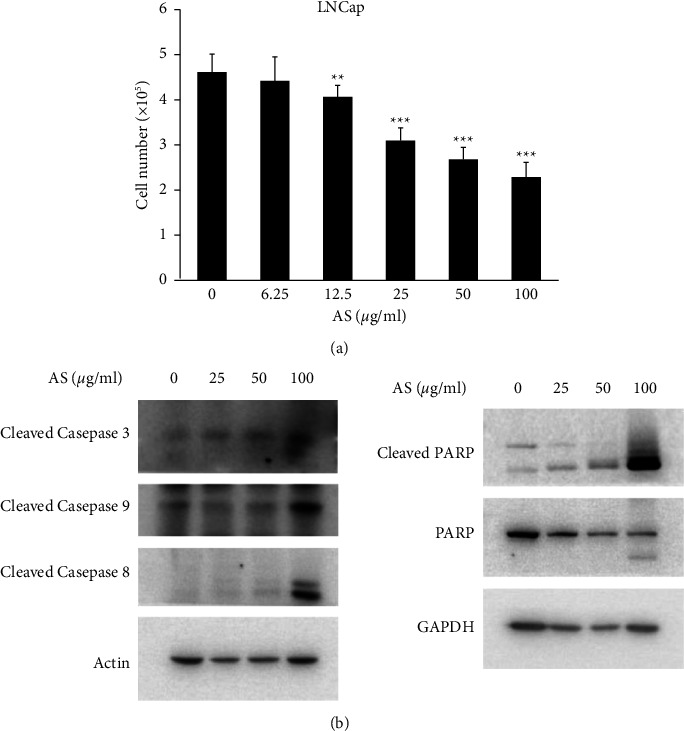
AS induces apoptosis of LNCaP cells. (a) LNCaP cells were seeded in 12-well plates and then treated with 6.25, 12.5, 25, 50, and 100 *μ*g/ml AS for 24 hours followed by trypan blue assay. Data were presented as the mean ± S.E.M. (Standard error of the mean) with three independent experiments (*n* = 3) and paired Student's *t*-test was used to calculate the *p*-value. Statistical significance was marked as ^*∗*^*p* < 0.05, ^∗∗^*p* < 0.01, and ^∗∗∗^*p* < 0.001. (b) LNCaP cells were seeded in 12 well plates and then treated with AS (0, 25, 50, and 100 *μ*g/ml) for 24 hours. Protein expression of apoptotic markers was evaluated by western blot with the specific primary antibodies anticleaved PARP, anti-PARP, anticleaved caspase 3, anticleaved caspase 8, anticleaved caspase 9, anti-GAPDH, and antiactin. GAPDH and actin were served as internal controls.

To verify whether AS extracts reduce the cell viability of the androgen-dependent prostate cancer LNCaP cell line, we performed a trypan blue assay accounting for the cell number to estimate the viability of LNCaP cells after AS treatment. The results demonstrated that AS treatment significantly decreased the cell viability in a dose-dependent manner (0, 6.25, 12.5, 25, 50, and 100 *μ*g/ml for 24 hours) in LNCaP cells (Figure 1). In addition, we assessed the protein expression levels of cleaved caspase 3, caspase 8, caspase 9, poly (ADP-ribose) polymerase (PARP), and cleaved PARP to determine the effect of AS treatment on the apoptotic pathways in LNCaP prostate cancer cells. The data showed that AS increased the protein levels of cleaved PARP and cleaved caspase 3/8/9 in LNCaP cells in a dose-dependent manner after 24 hours of treatment (Figure 1(b)). The expression and activation of apoptosis-related proteins in LNCaP cells indicated that the inhibition of LNCaP cell growth by AS should be related to apoptosis.

**Figure 2:**
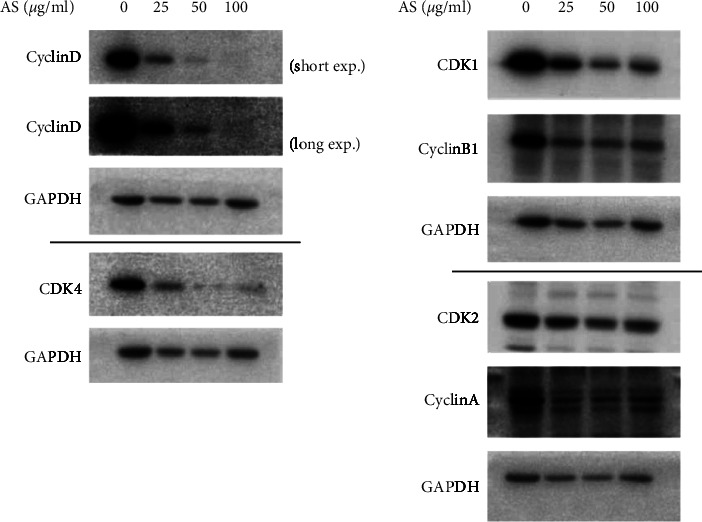
AS reduces the protein levels of CDK1, CDK2, and CDK4 and Cyclin B1, Cyclin A, and Cyclin D in LNCaP cells. LNCaP cells were seeded in 12-well plates and then treated with AS extracts (0, 25, 50, and 100 *μ*g/ml) for 24 hours. Western blot analysis was performed to evaluate the protein expression of cell cycle regulators with specific primary antibodies targeting CDK1, CDK2, CDK4, Cyclin A, Cyclin B1, and Cyclin D. GAPDH was served as an internal control.

To identify the cellular mechanism of AS-induced apoptosis, we evaluated the effect of AS treatment on the expression levels of cell cycle-related molecules in LNCaP cells. The results demonstrated that AS decreased the protein expression of CDK4/Cyclin D, CDK2/Cyclin A, and CDK1/Cyclin B1, which are G1, S, and G2 phase cell cycle regulators, respectively, in a dose-dependent manner in LNCaP cells (Figure 2).

**Figure 3:**
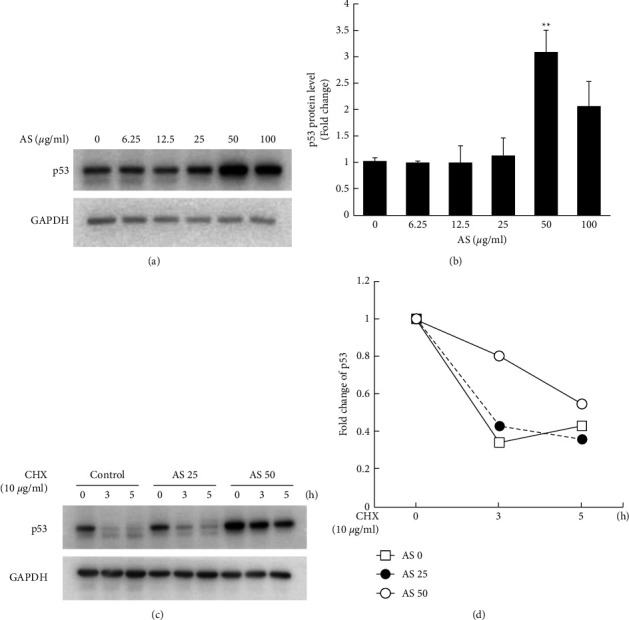
AS increases the protein level of p53 and reduces its protein degradation. (a) LNCaP cells were seeded in 12-well plates and followed with the treatment of AS with a series of concentrations (0, 6.25, 12.5, 25, 50, and 100 *μ*g/ml) for 24 hours. Western blot analysis was performed to identify the protein levels of p53 after AS treatment, and the protein level of GAPDH was served as an internal control. (b) The quantitative results of (a) were shown. (c) LNCaP cells were seeded in 12-well plates and then treated with AS (0, 25, and 50 *μ*g/ml) for 24 hours. After that, the cells were treated with 10 *μ*g/ml cycloheximide for 0, 3, and 5 hours before protein extraction. Western blot analysis was then performed to evaluate the protein level of p53 with specific p53 antibody. (d) The quantitative data of the p53 degradation curves were shown and the original p53 protein levels of all groups were adjusted as 1. Data were presented as the mean ± S.E.M. (Standard error of the mean) with three independent experiments (*n* = 3) and the paired Student's *t*-test was used to calculate the *p*-value compared to group = 0. Significance was marked as ^*∗*^*p* < 0.05, ^*∗∗*^*p* < 0.01, and ^*∗∗∗*^*p* < 0.001.

p53 is a negative regulator of cell cycle and its expression is accompanied by changes in the performance of other cell cycle regulators. According to the above results, AS treatment suppressed the expression of cell cycle regulators, including particular CDKs and cyclins (Figure 2). Therefore, we analyzed the protein level of p53 in LNCaP cells after treatment with a series of concentrations of AS extracts for 24 hours. The results indicated that AS treatment increased the protein level of p53 in LNCaP cells (Figures 3(a) and 3(b)). We further evaluated whether AS increases p53 level through modulating the protein stability of p53 in LNCaP cells. Cycloheximide, a protein synthesis inhibitor, was added into the culture medium 0, 3, and 5 hours prior to protein extraction under 24 h-AS treatment. The results in Figures 3(c) and 3(d) showed that after protein synthesis was blocked, the degradation of the p53 protein was significantly slowed down under AS treatment, and the p53 level remained higher than that in the control group, which may imply that the increasing p53 protein caused by AS is at least partially due to the enhanced protein stability of p53 in LNCaP prostate cancer cells.

**Figure 4:**
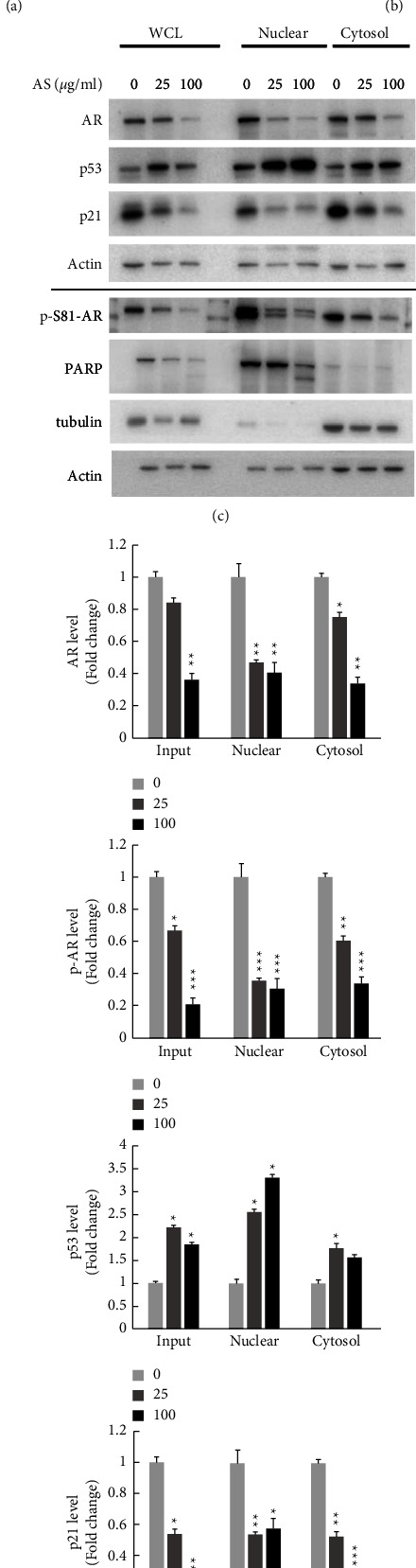
AS reduces expression and phosphorylation of AR, and causes nuclear localization of p53. (a) LNCaP cells were seeded in 12-well plates and then treated with AS (0, 6.25, 12.5, 25, 50, and 100 *μ*g/ml) for 24 hours. Western blot analysis was performed to evaluate the protein levels of AR phosphorylation at Ser-81 and total AR in LNCaP cells. (b) The quantitative results of (a) were shown, where actin was used as an internal control. (c) LNCaP cells were treated with AS (0, 25, and 100 *μ*g/ml) for 24 hours. Protein extraction was performed by nucleus/cytosol cell fractionation. Protein levels were detected by western blotting with specific antibodies targeting AR, p-s81-AR, p53, p21, and PARP. Actin protein was served as an internal control. PARP and tubulin were respectively served as nuclear and cytosolic markers. (d) The quantitative results of (c) were shown. Data were presented as the mean ± S.E.M. (standard error of the mean) with three independent experiments (*n* = 3) and the paired Student's *t*-test was used to calculate the *p*-value compared to group = 0. Significance was marked as ^*∗*^*p* < 0.05, ^∗∗^*p* < 0.01, and ^∗∗∗^*p* < 0.001.

AR is known to play an essential role in prostate cancer cell proliferation [17]. Our previous study has shown that the specific phosphorylation site of AR on Ser-81 is the major regulator for AR activation, which increases the stabilization of AR protein in the nucleus [4]. On the other hand, p53 is reported to be involved in androgen signaling, and the overexpression of wild-type p53 depresses androgen function in both LNCaP and 22Rv1 human prostate cancer cell lines [16]. Accordingly, we hypothesize that AS treatment might suppress the protein expression and stability of AR, based on the observation of increased p53 protein expression and stabilization following AS treatment in LNCaP cells (Figure 3). The results showed that AS treatment significantly decreased the levels of both p-S81-AR and AR proteins and also the ratio of p-S81-AR to AR proteins (p-S81-AR/AR) in a dose-dependent manner (Figures 4(a) and 4(b)), accompanied by an increase in p53 protein expression in LNCaP prostate cancer cells (Figure 3(a)). In addition, AS treatment significantly inhibited the distribution of both p-S81-AR and total AR in the nucleus and cytoplasm, while increasing the nuclear and cytosolic distribution of p53 (Figure 4(c)). Quantitative data also showed that AS significantly reduced p-S81-AR in all cell fractions, especially in the nucleus (Figure 4(d)). These results revealed that AS decreased p-S81-AR level, thereby reducing its stability and resulting in a decrease in AR protein expression. The decrease in AR was accompanied by an increase in p53 protein levels in LNCaP cells, suggesting that the negative regulation of AR by p53 might be triggered after AS treatment.

**Figure 5:**
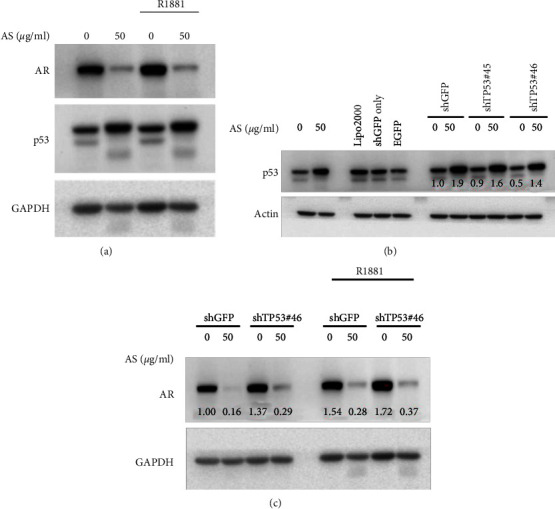
AS inhibits AR through p53 upregulation. (a) LNCaP cells were seeded in 12-well plates and then treated with AS (0, 50 *μ*g/ml) and 0.1 nM R1881 for 24 hours. (b) The expression of p53 was evaluated following knockdown of p53 by shRNA. (c) Knockdown of p53 was performed by shTP53 in LNCaP cells in the presence of R1881 (a synthetic androgen; 0.1 nM for 24 hours). The control groups were transfected with shGFP. Protein levels were detected by western blotting analysis with specific AR and p53 antibodies. GAPDH served as internal controls.

The above observations showing that AS treatment increased the expression of p53 in LNCaP cells, especially in the nucleus, and downregulated AR expression and phosphorylation prompted us to further evaluate whether AS-mediated AR suppression is controlled by p53. A synthetic androgen agonist R1881, also known as metribolone, was used to stimulate AR under the treatment of AS extracts. The results showed that, in the absence of AS treatment, the addition of R1881 increased the AR protein level in LNCaP cells. However, AS treatment inhibited AR protein level while enhancing p53 protein expression with or without the addition of R1881 (Figure 5). These data indicated that R1881 had less effect on AR protein stability under AS treatment. Accordingly, we supposed that the AR inhibition caused by AS is mediated through p53 activation. To clarify that, we knocked down p53 by shRNA transfection in the presence of R1881 in LNCaP cells. The results showed that inhibition of p53 enhanced AR protein levels under AS treatment in the presence of R1881. The results demonstrated that suppression of AR caused by AS treatment is mediated by p53 upregulation (Figure 5(c)).

*Antrodia cinnamomea* (AC) is an endemic fungal species in Taiwan, which has an essential health promotion activity against numerous diseases, including cancer, hypertension, abdominal pains, and diarrhea [19, 24, 25]. AC has been reported to have pharmacological efficacy against a variety of cancer types [26]. Antrocin, a sesquiterpene lactone isolated from *A. cinnamomea*, has been shown to have a synergistic inhibitory effect on conventional therapies to inhibit proliferation and induce apoptosis in cancer cells. In addition, the combination treatment of antrocin and ionizing radiation (IR) induces cell cycle arrest at the G2/M phase and triggers apoptosis in radioresistant prostate cancer cells [27]. However, the AC is an endangered endemic species in Taiwan. It is rare and precious because it can only grow on the inner surface of the heartwood cavity of the evergreen tree Cinnamomum kanehirai Hayata (Lauraceae) [28]. Using it as a medicinal material will face supply and demand or ecological issues such as difficulty in obtaining and conservation of species. A closely related species, *Antrodia salmonea* (AS), which grows on the indigenous coniferous tree Cunninghamia konishii Hayata (Cupressaceae), has recently been reported to have anticancer effects as AC does. It has been shown that AS inhibits cancer cell growth and induces cell apoptosis in human triple-negative breast cancer [29], ovarian carcinoma cells [8], and human promyelocytic leukemia [30]. Since AS has been shown to have beneficial effects in the treatment of multiple diseases, including inhibition of cancer cell growth, AS is considered an alternative to *A. cinnamomea* and can preclude cost considerations due to its market rarity. In addition, it can avoid the overcollection of rare *A. cinnamomea*, which is on the verge of extinction.

Recent studies have demonstrated that AS induces G2 phase cell cycle arrest and inhibits the cell cycle-related proteins Cyclin A, Cyclin B1, Cyclin D, Cyclin E, and CDK2 in human triple-negative breast cancer [29] and G1 phase arrest in human promyelocytic leukemia [30]. Accordingly, we evaluated the anticancer effect of the extract of AS on prostate cancer cells in this study. Our current data showed that AS treatment significantly inhibited cell viability in LNCaP cells. Furthermore, we assessed the role of AS in the regulation of the cell cycle in prostate cancer cells. The results indicated that AS treatment inhibited cell cycle regulators including CDK1, CDK2, CDK4, Cyclin B1, and Cyclin D in LNCaP prostate cancer cells. These results are consistent with previous studies and also imply that AS causes cell cycle arrest and finally apoptosis by inhibiting the expression of cell cycle regulators.

AR is crucial for the survival of LNCaP cells. Any form of AR inhibition can affect the viability and even lead to apoptosis in LNCaP cells [31]. AR degradation has even recently become an important strategy for the treatment of prostate cancer [32]. On the other hand, decreased phosphorylation of AR on S81-site is also an important indicator for inhibiting AR and prostate cancer cell proliferation [33]. Therefore, in our study, AS-induced apoptosis in LNCaP cells is most likely related to the significant reduction of AR protein through decreasing p-S81-AR.

Wild-type p53 is crucial for AR regulation, which has a protective effect on AR in androgen signaling. Overexpression of wild-type p53 has been shown to decrease AR function in both LNCaP and 22Rv1 cells [12, 34]. Studies have shown that p53 and AR may negatively regulate each other. For instance, knockdown of p53 increases AR protein expression level [15], while activation of p53 leads to AR protein destabilization [35]. In addition, loss of p53 induces the AR-mediated oncogenic transformation [36], aggressiveness, and trans-differentiation in prostate cancer progression [37]. These findings indicate that p53 and AR have a functional interaction and provide evidence for the importance of p53-mediated AR signaling in the carcinogenesis of human prostate cancer cells. According to the above, we evaluated the protein expression of p53 and its regulation on AR protein level in androgen-sensitive LNCaP prostate cancer cells. Our data showed that AS treatment significantly increased the protein stability of p53 in androgen-sensitive LNCaP prostate cancer cells (Figure 3). Subsequently, we evaluated the protein level of AR along with p53 and their subcellular distribution in the nucleus and cytosol. Our data revealed that AS significantly reduced phospho-AR (Ser81) and total AR levels in a dose-dependent manner (Figure 4). Inhibition of AR was accompanied by a marked increase in nuclear p53 protein in LNCaP cells and a mild increase in cytosolic p53. These data suggest that AS treatment in androgen-sensitive prostate cancer cells manipulates the interaction of p53 and AR signaling and affects their activity and signaling, thereby inhibiting cell survival and inducing apoptosis. In other words, AS treatment may have a toxic effect on androgen-sensitive prostate cancer cells through negative regulation between p53 and AR.

Our recent studies have confirmed that AS disrupts EGFR-AKT and EGFR-ERK signaling pathways and inhibits the growth of androgen-independent prostate cancer cell lines DU145 and PC3. The increase of p21 and p27 proteins in the nucleus arrests cell cycle progression and leads to apoptosis in DU145 and PC3 cells following AS treatment [37]. DU145 and PC3 cell lines express very low levels of AR [38] and mutated p53 [39, 40], in contrast to the LNCaP cell line, which is androgen-sensitive and expresses a higher level of AR and wild-type p53. Although AS suppresses cancer cell growth and induces apoptosis in androgen-independent prostate cancer DU145 and PC3 cell lines as well as the androgen-sensitive LNCaP cell line. AS treatment in LNCaP depressed p21 levels in total cell lysate and nuclei, in contrast to the effect of AS treatment on p21 protein in DU145 and PC3 cells. The p53 protein level in DU145 cells was not affected following AS treatment (Figures S1), whereas p53 was elevated in the LNCaP nuclear fractions. The increase of p53 disrupted AR stability and expression and arrested cell cycle progression in LNCaP cells, which thus blocked cell growth and led to apoptosis. Although the different mechanisms of AS-induced growth inhibition in prostate cancer cells have been identified whether EGFR signaling participates in AS-induced growth inhibition in LNCaP cells or whether other signaling is involved, remains to be further evaluated.

Taken together, we infer that the anticancer effects of AS in prostate cancer may be mediated through multiple pathways, in response to the expression profiles of AR and p53 as well as the androgen-dependent properties of prostate cancer cells.

## 4. Conclusions

**Figure 6:**
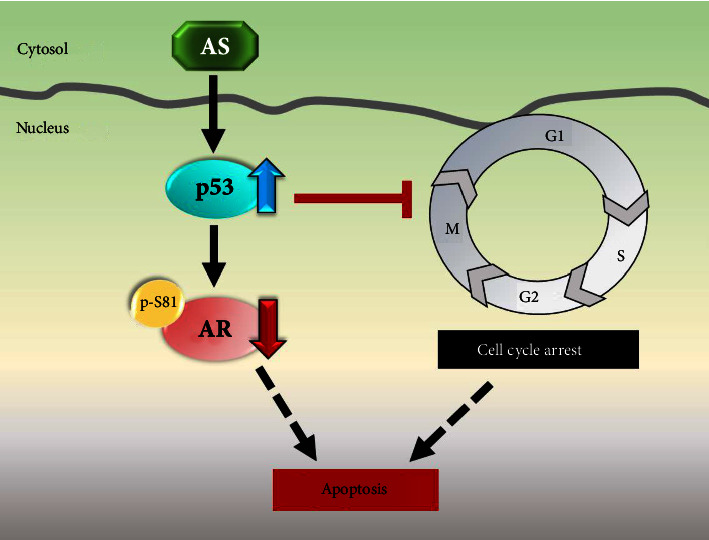
Schematic diagram of AS-mediated regulation of apoptosis in prostate cancer. AS enhances p53 protein stability and nuclear accumulation in androgen-dependent human prostate cancer cells. Increased p53 inhibits both the Ser-81 phosphorylation and protein stability of AR in the nucleus. Decreased protein expression/activation of AR through p53 upregulation, therefore, causes apoptotic features in prostate cancer cells. Besides, AS-mediated p53 activation results in cell cycle inhibition. Overall, AS inhibits prostate cancer cell proliferation through p53 regulation and AR inhibition.

In conclusion, the findings in the present study suggest that *A. salmonea* suppresses cell cycle progression and induces apoptosis, thereby inhibiting the viability of androgen-sensitive prostate cancer cells through activating p53 and inhibiting androgen receptor signaling in the nucleus (Figure 6). Overall, our current study provides a new approach to the treatment of human prostate cancer by using a healthy fungus, A. salmonea. This also provides a solution in replacing endangered AC with AS to develop new treatments for cancer.

## Data Availability

The data used to support the findings of this study are available from the corresponding author upon request.

## Supplementary Materials

Supplementary Materials Supplementary data (Figure S1) indicated the protein level of p53 in DU145 prostate cancer cells following a series doses of AS treatment. Figure S1: The protein level of p53 in DU145 cells after AS treatment. DU145 prostate cancer cells were treated with a series of AS doses, as indicated. Western blot analysis was performed to identify the protein levels of p53 after AS treatment for 24 h and the protein level of GAPDH was served as an internal control.
