# Supplementary material for: Antrodia salmonea Extracts Regulate p53-AR Signaling and Apoptosis in Human Prostate Cancer LNCaP Cells
Source: Evid Based Complement Alternat Med. 2022 Nov 29;2022:7033127. doi: 10.1155/2022/7033127 (PMC9726253; doi:10.1155/2022/7033127)
Supplement: Supplementary Materials — Supplementary data (Figure S1) indicated the protein level of p53 in DU145 prostate cancer cells following a series doses of AS treatment. Figure S1: The protein level of p53 in DU145 cells after AS treatment. DU145 prostate cancer cells were treated with a series of AS doses, as indicated. Western blot analysis was performed to identify the protein levels of p53 after AS treatment for 24 h and the protein level of GAPDH was served as an internal control. [file 7033127.f1.pdf]

Figure S1. The protein level of p53 in DU145 cells after AS treatment.

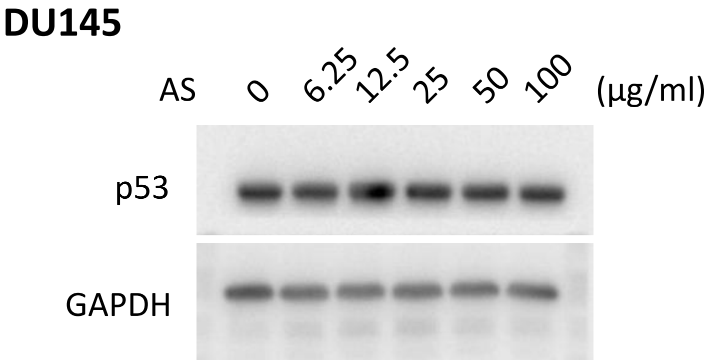

Figure S1. The protein level of p53 in DU145 cells after AS treatment. DU145 prostate cancer cells were treated with a series doses of AS, as indicated. Western blot was performed to identify the protein levels of p53 after AS treatment for 24 h, and the protein level of GAPDH was served as an internal control.
